# Supplementary material for: Rapid Direct Nucleic Acid Amplification Test without RNA Extraction for SARS-CoV-2 Using a Portable PCR Thermocycler
Source: Genes (Basel). 2020 Jun 18;11(6):664. doi: 10.3390/genes11060664 (PMC7349311; doi:10.3390/genes11060664)
Supplement: Supplementary file 1 [file genes-11-00664-s001.pdf]

genes

MDPI

Supplementary Information S1

Article

Rapid Direct Nucleic Acid Amplification Test Without RNA Extraction for SARS-CoV-2 Using a Portable PCR Thermocycler

Soon Keong Wee, Suppiah Paramalingam Sivalingam and Eric Peng Huat Yap *

1. Protocol for DIRECT-PCR of SARS-CoV-2 from crude respiratory samples

This protocol describes the single-tube one-step DIRECT-PCR protocol of SARS-CoV-2 from crude respiratory samples. It details the preparation of DIRECT-PCR mastermix and sample processing.

2. Materials and Methods

Table S1. Consumables used for the direct detection of SARS-CoV-2.

Reagents and Consumables	Storage Condition
10 µM Primers and Probes	−30 °C freezer
Invitrogen™ RNaseOUT™ Recombinant Ribonuclease Inhibitor (5000 U, Specific activity: 40 U/µL, ThermoFisher Catalog 10777-019)	−30 °C freezer
VitaNavi Technology Direct One-Step S/P RT-qPCR TaqProbe Kits (No Reference Dye)<br>VitaNavi 2X S/P RT-qPCR TaqProbe Mix<br>VitaNavi S/P RT-qPCR Pols	−30 °C fridge
Thermo Scientific™ Remel™ Sputasol (Oxoid)	4 °C fridge
Molecular grade DNase/RNase-free water	4 °C fridge
General Consumables:<br>Micropipette<br>Aerosol-resistant tips<br>Centrifuge<br>Vortex<br>1.5 mL Microcentrifuge Tube<br>qPCR plate<br>qPCR plate optical adhesive film<br>PCR 8-strip tubes and caps	Room Temperature

3. Primers and Probes

Table S2. Primers and Probes used for the direct detection of SARS-CoV-2.

Target	Primer Name	Sequence (5′ to 3′)	Amplicon size	Reference
Human RNase P internal control	RP-F	AGATTTGGACCTGCGAGCG	65 bp	(CDC, 2020)
	RP-R	GAGCGGCTGTCTCCACAAGT		
	RP-Probe	FAM–TTCTGACCTGAAGGCTCTGCGCG–BHQ1		
SARSCoV-2 ORF1ab	SARS-CoV2_ORF1ab-F	CCCTGTGGGTTTTACACTTAA	119 bp	(C. CDC, 2020; Wang et al., 2020)
	SARS-CoV2_ORF1ab-R	ACGATTGTGCATCAGCTGA		
	SARS-CoV2_ORF1ab-probe	FAMCCGTCTGCGGTATGTGGAAAGGTTAT GG-BHQ1		
SARSCoV-2 N	SARS-CoV-2_N-F	GGGGAACTTCTCCTGCTAGAAT	99 bp	
	SARS-CoV-2_N-R	CAGACATTTTGCTCTCAAGCTG		
	SARS-CoV-2_NProbe	FAM-TTGCTGCTGCTTGACAGATTTAMRA		

Preparation of Mastermix Area

1. Set up separate physical areas for mastermix reagent preparation, clinical sample preparation, and post-amplification area.
2. Disinfect all work surfaces with appropriate disinfectant.
3. Establish a unidirectional workflow to reduce contamination.
4. Do not mix materials or equipment from the sample preparation area and reagent preparation area.
5. Use separate set of pipettes are available for use in each area.
6. Use aerosol resistant tips to prevent aerosols and reduce contamination.
7. Always maintain ice box or cooler box for use to prepare reagents and samples in cold chain.

PCR Mastermix Preparation

Table S3. Concentration and volume of PCR mastermix reagents for 10 µl reaction.

Reagent	Final Conc.	Volume (µl) per rxn
VitaNavi 2X S/P RT-qPCR TaqProbe Mix	1X	5.0
VitaNavi S/P RT-qPCR Pols	1X	0.5
Invitrogen™ RNaseOUT™ Recombinant Ribonuclease Inhibitor	0.8 U/µl	0.2
10 µM Forward primer	400 nM	0.4
10 µM Reverse primer	400 nM	0.4
10 µM FAM-Probe	200 nM	0.2
Template	-	1.0 to 2.0
Molecular grade H20	-	Adjust the volume accordingly
Total	-	10.0

PCR Mastermix Preparation

1. Bring all reagents on ice (Table S3).

2. Flick the reagents to mix before use.

3. Include PCR positive template control sample and a non-template control (NTC) in each run

4. Prepare separate Mastermix for each primer set (SARS-CoV-2 N gene, SARS-CoV-2 ORF1ab gene and human RP gene)

5. Label 1.5 mL microcentrifuge tube as MasterMix.

6. Add all the components in the following order. (a) Add water first. (b) Add forward and reverse primers. (c) Add TaqProbe mix buffer. (d) Add probes. (e) Add RNaseOUT enzyme (f) Add VitaNavi S/P RT-qPCR Pols enzyme last.

7. Mix the Mastermix by inverting the tube at least 10 times.

8. Aliquot Mastermix equally into qPCR plate/tubes (e.g. for 1 µL template, add 9 µL of Mastermix)

9. Cover the plate with aluminium foil to minimize probe exposure to light.

Clinical Sample Preparation

1. All clinical specimens must be carried out in a Class II Biological Safety Cabinet (BSC) in Biosafety Level-2 (BSL-2+) Plus Laboratory in accordance to local authority guidelines.

2. Setup Class II Biological Safety Cabinet (BSC).

3. Heat inactivate respiratory samples such as sputum or nasal, throat, nasopharyngeal swabs at 70°C for 5 minutes immediately upon receiving them.

4. Aliquot 1 mL of the samples into a sterile tube.

5. Pre-prepare working concentration Sputusol (Oxoid) by adding entire vial content (7.5 mL) to 92.5 mL of sterile water accordingly to manufacturer's instruction.

6. Add 1 mL of Sputasol (in working concentration) to mix with the sample. The ratio of sample : sputasol is 1:1 (v/v).

7. Vortex for 5 minutes.

Addition of templates into MasterMix

1. Add 1 to 2 µL of samples into the Mastermix well without introducing bubbles. Note: This brings the final PCR reaction volume to 10 µL.

2. Add 1 µL of RNA positive control sample into the positive control well.

3. Add 1 µL of non-template control (NTC) sample into the negative control well.

4. Seal the qPCR strip-tube with the optical cap / qPCR plate with the optical adhesive film.

5. Briefly centrifuge (spin-down) the qPCR mix and ensure that there are no bubbles.

PCR Protocol

1. Set up the qPCR Machine according to manufacturer's instructions. a. Example: Comparative CT mode, 96-well, Taqman, fast ramp rate, FAM-Probe, No quencher

2. Setup the dRT-qPCR run condition as follows: Reverse Transcription: 50°C – 5 minutes, Initial denaturation: 95°C - 30s, Cycling stage: 40 x (95°C - 10s + 55°C - 15s)

Analysis of Results

1. After completion of the run, save and analyze the experimental data following the instrument manufacturer's instructions.

2. Analyses should be performed separately for each target using a manual threshold setting.

3. Thresholds should be adjusted to fall within exponential phase of the fluorescence curves and above any background signal. The procedure chosen for setting the threshold should be used consistently.

4. Negative for NTCs (with water) must be blank.

5. Positive controls must be positive with < 40 Cq value.

6. Internal RNase P controls for clinical samples must be positive with < 40 Cq value.

7. If SARS-CoV-2 N gene and ORF1ab gene crosses the cycle threshold line within 40 Cq, the samples will be considered positive for Covid-19 infection.

© 2020 by the authors. Submitted for possible open access publication under the terms and conditions of the Creative Commons Attribution (CC BY) license (http://creativecommons.org/licenses/by/4.0/).
